# An integrated remediation approach using combinations of biochar, *Rhizobium leguminosarum*, and *Vigna radiata* for immobilizing and dissipating cadmium contaminants from the soil–mustard plant system

**DOI:** 10.3389/fpls.2023.1139136

**Published:** 2023-03-06

**Authors:** Qurat-ul-Ain Ali Hira, Midhat Mahboob, Rimsha Azhar, Faiza Munir, Alvina Gul, Asim Hayat, Tariq Shah, Rabia Amir

**Affiliations:** ^1^ Department of Plant Biotechnology, Atta-ur-Rahman School of Applied Biosciences (ASAB), National University of Sciences and Technology (NUST), Islamabad, Pakistan; ^2^ Land Resource Research Institute, National Agricultural Research Center (NARC), Islamabad, Pakistan; ^3^ Plant Science Research Unit, U.S. Department of Agriculture-Agricultural Research Service (USDA-ARS), Washington, DC, United States

**Keywords:** cadmium remediation, mustard plant, phytoremediation, biochar, intercropping, plant growth-promoting rhizobacteria (PGPR)

## Abstract

Cadmium (Cd) contamination of soils is an environmental concern, as cadmium harms food crops and can therefore impact human health. The use of combinations of biochar (seeded with *Rhizobium leguminosarum*) and *Vigna radiata* (as an intercrop) has the potential to reduce the mobilization of Cd from soil *via* mustard plants (*Brassica juncea*). Mustard plants are grown as a food and oil production crop that is consumed worldwide. However, this plant has the property of hyperaccumulation; thus, it bioaccumulates Cd in its tissues, which in turn, if eaten, can become part of the human food chain. Hence, reducing Cd bioaccumulation in mustard plants is crucial to making these plants a reliable and safe source of food for consumption. To improve soil sorption capacity and immobilization efficiency, biochar (in the form of wheat husk) was mixed with *R. leguminosarum* and intercropped (using *V. radiata)* with mustard plants for further investigation. Sampling was performed at an early growth stage (i.e., at 30 days) and at maturity (i.e., at 60 days) to determine the impact of Cd on a plant’s morphophysiological attributes. Data were analyzed in two ways: first by analysis of variance (ANOVA) and then by the *post hoc* Tukey’s honestly significant difference (HSD) test. The statistical analysis concluded that combinations effectively improved plant traits by 65%–90% in the early growth stage and by 70%–90% in the maturity stage. The T6 treatment combination [i.e., biochar + *R. leguminosarum* + *V. radiata* (BC + RL + VR)] provided the most effective results in terms of growth, biomass, pod yield, and pigmentation content. In addition, this combination reduced the translocation of Cd in mustard plants by 70%–95%. The combination of BC + RL + VR effectively reduced Cd contamination of mustard tissue and provided a suitable growing environment for the plants. A post-harvesting soil analysis using X-ray diffraction (XRD) found that Cd was undetectable in soil. This provides clear confirmation that these approaches can lead to Cd soil remediation. Moreover, this study provided insight into the responses of different morphophysiological attributes of mustard plants to Cd stress and could aid in developing Cd stress tolerance in mustard plants.

## 1 Introduction

Rapid industrialization has harmful environmental effects resulting from the production of pollutants, including heavy metals (HMs). HMs are not essential elements for plant growth; consequently, HMs harm the growth and development of plants (Anjum et al., 2012). Cadmium (Cd) is one HM that can disturb the mechanisms of the whole plant, which in turn leads to plant death, and is considered the seventh most toxic HM in food crops. Cd persists in soil for long periods of time because of its simple structure and molecular weight. A soil Cd concentration ranging from > 5 to 10 mg/kg is considered toxic for plant growth (Mutlu et al., 2012). Cd travels through the xylem and accumulates in the aerial parts of plants. As leaves are considered the functional factories of plants, the accumulation of Cd in leaves disturbs all plant processes, including water and nutrient uptake and the manufacture of photosynthetic pigments (Da-lin et al., 2011). On a morphological level, the toxic effects of Cd manifest as reductions in plant growth, biomass, and yield quantity, increasing root length, and structural changes in the roots (Khan et al., 2007; DalCorso et al., 2008; Hossain et al., 2009; Hossain et al., 2010; Da-lin et al., 2011; Rascio and Navari-Izzo, 2011; Villiers et al., 2011).

Specific plants that have the strategies to adapt to Cd stress and have the ability to continue to grow and survive in this type of stress are known as hyperaccumulators. *Brassica juncea* (mustard plant) belongs to the family Brassicaceae (also known as Cruciferae) and is known as a hyperaccumulator plant. It is an important vegetable and oil crop, containing significant quantities of antioxidant and anti-inflammatory compounds, vitamins, and nutrients (Vélez, 2017). Mustard plants have the property of being able to accumulate Cd in their leaves, which in turn causes stunted growth (Kapoor et al., 2014). Green strategies (i.e., phytoremediation) also use mustard plants to bioremediate Cd-contaminated soils. Unfortunately, the green strategy also has some limitations, as most hyperaccumulating plants show growth retardation, low yield, and minimal economic value. Mustard plants that are used for phytoremediation programs, which have low yield quality, are not acceptable for food and oil production; however, these plants can be converted into beneficial products, such as biochar and biofuel, and be used in fermentation and ethanol production processes (Ye-Tao et al., 2012; Dhiman et al., 2016; ur Rehman et al., 2017).

Various plants and crops cannot survive under Cd stress; for this reason, scientists are working to develop biological approaches that can effectively overcome Cd toxicity, including intercropping, bioremediation using microbes, and soil amendments using biochar (Tang et al., 2020; Houssou et al., 2022; Taye et al., 2022). Intercropping is a method of cultivation of two or more crop species in the same field, and it can enhance plant productivity and biodiversity and provide biocontrol against pests and pathogens. Intercropping is also a suitable agricultural management practice for improving the phytoremediation process in brassicaceous plants (Cao et al., 2019). The selection of plant species for intercropping is key to the successful implementation of strategies that enhance the translocation of Cd from the roots to the shoots in mustard plants (Wang et al., 2019). Leguminous plants solubilize insoluble phosphorus and fix nitrogen through biological processes in the soil (Etemadi et al., 2019). Couëdel et al. (2018) confirmed that leguminous plants accumulate nitrogen in plant tissues under intensive HM stress. Mung beans (*Vigna radiata* L.) belong to the legume family “Fabaceae” and this plant species is known to be able to survive and grow under Cd stress. Wu et al. (2019) confirmed that intercropping of mung bean with tamarillo (*Cyphomandra betacea*) plants increases the defense mechanism in plants grown under Cd stress. However, to our knowledge, no extensive study on the intercropping of mung beans has been reported and conducted until now.

Many plant-beneficial soil microbes have been used for the bioremediation of Cd-contaminated soil. The rhizosphere plant root system closely interacts with soil microbes (Berg and Smalla, 2009). Plant–microbe interactions have provided potential evidence against Cd stress in various studiesSeveral studies have provided evidence that plant–microbe interactions can potentially mitigate the effects of Cd stress (Lay et al., 2018; Cordero et al., 2020; Taye et al., 2020). The *Rhizobiaceae* bacterial family is known for its symbiotic relationship with leguminous plants, which are capable of nitrogen fixation. A study by Noel et al. (1996) shows that *Rhizobium leguminosarum*’s symbiotic interaction with *Brassica napus* significantly enhances root length. Taye et al. (2022) confirmed the positive influence of *R. leguminosarum* on root architecture, plant health, and productivity of *B. napus*. Despite the importance of *B. juncea* root architecture, root growth dynamics and the relationship between root system architecture and microbe–cadmium interactions are still unknown. This was also a major focus of our study.

Different biological techniques have been investigated as a means to reduce Cd toxicity in agricultural land. The use of biochar (BC) has been widely reported as a means to increase the tolerance of crops to HM stress (Ahmad et al., 2016). BC products have a porous structure with high surface areas and absorption properties; consequently, these products enhance the ability of the plant to absorb and bioaccumulate various HMs and organic contaminants from the surrounding soil. BC also provides nutrients and minerals to plants, resulting in the enhancement of plant growth parameters (Younis et al., 2016). However, very little information is available regarding the effect of BC products on the morphophysiological and biochemical attributes of the Brassicaceae family, especially when grown under Cd stress.

The primary objective of this study was to find the best combination of BC product, co-planting (i.e., intercropping plant species), and bacterial strain that would improve soil structure to reduce the immobilization of Cd and confer CD tolerance on mustard plants while at the same time enhancing mustard morphophysiological traits. For this purpose, the variety BARD1 of *B. juncea* was selected based on the yield traits, fast growth progression, and high Cd tolerance. Thus, to prevent Brassica Juncea crop growth and yield quantity under Cd stress. Some co-combination techniques have been adapted to analyze their mutual benefits comprising toward the crop, including the bacterial strain of species (Rhizobium leguminosarum), biochar produced from (Wheat husk), and Co-planting with legume plant (Vigna radiata). This study also provides data to investigate the root architecture of plants grown under Cd stress (Nedjimi and Daoud, 2009; Huang et al., 2010; Houssou et al., 2022).

## 2 Materials and methods

### 2.1 Seed collection and germination

Mustard plant seeds were collected from the Oil & Seed Research Program, National Agriculture Research Center (NARC), Pakistan. This department preserves the germplasm of oilseed crops and, using hybridization, evaluates oil plant varieties for enhancement of yield; heat, pest, and drought resistance; and nutritional qualities. Four varieties of mustard plants were used for the germination rate test: BARD1, Canola, Bahawalpur, and Super-Raya. BARD1 was selected as this variety showed an 85% germination rate (Sharma and Bhardwaj, 2007). Experiments using the selected variety were conducted at Atta ur Rehman School of Applied Bioscience, NUST, Pakistan. Seeds were surface sterilized with 70% ethanol for 1 min, followed by washing with distilled water. Seeds were spread on filter paper inside a safety cabinet for maximum ethanol evaporation. UV-sterilized germination paper was used for seed germination in a box. The box was wrapped with aluminum foil to minimize light penetration. The box was placed in a dark place at 25°C–28°C for 48 hours to break seed dormancy (Alvarado and Bradford, 2005).

### 2.2 Seedling growth and experimental design

Soil with pH 7.78, which was autoclaved at 121°C for 1 hour, was used to fill the pots. Each pot contained 1.2 kg of soil. A 10 mmol/kg cadmium chloride (CdCl_2_) solution was added to each pot. Cadmium was added to the soil by watering it with the CdCl_2_ solution for 10 days. A total of 12 g of biochar (produced from wheat husk) was mixed into each pot, and 1 mL of the bacterial inoculum of *R. leguminosarum* was transferred to the soil 3 days before the sowing of the seedlings. The glasshouse chamber had a 16-h day/8-h night photoperiod, with a light intensity of 300/μmol/m^2^/s. The temperature was set at 20°C, with a relative humidity of 50%–60%. Seedlings with a constant growth rate were planted in pots after the 10th day of treatment with the cadmium solution. The treatment design was as follows: negative control (NC), a single mustard plant; positive control (PC), mustard plant + cadmium; T1, *R. leguminosarum* (RL) inoculation only; T2, biochar only (BC); T3, *R. leguminosarum + *biochar; T4, *V. radiata* only (var. MN-11) (VR); T5, *V. radiata + R. leguminosarum*; T6, *V. radiata + R. leguminosarum + *biochar. The experimental pots were based on a completely randomized design with seven replicates, and data analysis was performed on six replicates. Plants were harvested for data analysis at two different time points, on day 30 day (flower/early-stage harvesting) and day 60 (pod/maturity stage).

### 2.3 Water-holding capacity

To determine the water-holding capacity of the soil, we added 1.2 kg of soil to each of the six pots: three contained plain soil (control group), and three contained a biochar mixture (experimental group). The three control pots were placed in one tub of water and the experimental pots in the other. Water was added to all six pots until it started dripping from the bottom. After 4 hours, pots were weighed with an electrical balance (*W*_1_). The soil was dried in the oven for 2 hours at 100°C, after which the soil was again weighed (*W*_a_). Air-dried pots were kept at room temperature and their weight was defined as *W*_b_. *W*_2_ was defined as *W*_2_ = *W*_a_ + *W*_b_. Water-holding capacity (WHC) was measured by the following formula (Wang et al., 2014):


$$100\%\;WHC=\;\frac{W_{1}+\;W_{2}}{W_{2}}*100$$

### 2.4 Morphological traits

Root, shoot, leaves, and pods [length and fresh and dry weights (g)] were measured during the first period (i.e., early stage of growth), on the 30th day, and at the end of the second period (i.e., mature stage), at harvesting, on the 65th day. Root and shoot length (cm) were measured manually using a measuring tape. The fresh weight was measured with a digital balance immediately after harvesting. The dry weight was collected by washing the roots, shoots, and leaves with deionized water, then the tissues were air dried in an air dryer at 70°C for 2 hours to remove moisture. The tissues were then weighed to determine the dry weight of the samples (Aina et al., 2019). Leaves and roots were scanned with ImageJ software (National Institutes of Health, Bethesda, MD, USA), leaves area was calculated in ImageJ software. However, for the measurement of various roots parameters GiARoots (general image analysis of roots) software was used for further analysis (Schneider et al., 2012). Fresh leaf samples from each plant were collected to calculate relative water content. Leaf samples were kept in water for 24 hours to allow them to regain turgidity. The samples were then weighed to determine the turgid weight. Leaves were then oven-dried at 70°C for 4 hours to determine the dry weight. The relative water content (RWC) was measured by using the formula described by Fariñas et al. (2019):


$$RWC=\frac{Fresh\;weight-dry\;weight}{turget\;weight-dry\;weight}\times 100$$

### 2.5 Root structure analysis

The root scanning method is considered effective for analyzing different parameters of roots. The plant root samples were washed with deionized water and then dried on filter paper until the overlapping of root hairs was observed. Sample roots were aligned on the Plastic sheet and scanned with a scanner. The images collected from the scanner were analyzed using the GiA Roots software (Tripathi et al., 2021).

### 2.6 Photosynthetic pigment analysis

The following method was used to measure the chlorophyll and carotenoid contents (Arnon, 1949). A 0.5-g sample of fresh plant tissue was homogenized with 5 mL of 80% acetone. The homogenized sample was centrifuged at 13,000 rpm for 20 min at 4°C. The supernatant of plant extract was collected for analysis. The absorbance of the supernatant was measured at 645, 663, and 470 nm for chlorophyll a, chlorophyll b, and carotenoid content, respectively. In the equations below, *A* represents the absorbance at a specific wavelength, *V* is the final volume of the chlorophyll extract in 80% acetone, and *W* is the fresh weight of the tissue extracted.


$$Chlorophyll\;A=\frac{12.7\left(A_{663}\right)-2.69\left(A_{645}\right)\times V}{1000}\times W$$

$$Chlorophyll\;B=\frac{22.9\left(A_{645}\right)-4.68\left(A_{645}\right)\times V}{1000}\times W$$

$$Total\;Chlorophyll\;=\frac{20.2\left(A_{645}\right)-8.02\left(A_{663}\right)\times V}{1000}\times W$$

$$Carotenoid\;=\frac{7.6\left(A_{470}\right)-1.49\left(A_{510}\right)\times V}{1000}\times W$$

### 2.7 Field-emission scanning electron microscopy and energy-dispersive X−ray spectrometric analysis for Cd

The surface morphology of dry root and leaf samples was monitored using field-emission scanning electron microscopy (FESEM) (model JSM 6490A; JEOL Ltd, Tokyo, Japan). The elemental composition of the tissues was analyzed using energy-dispersive X−ray (EDX) spectrometry (model JSM 6490A; JEOL Ltd). For the analysis, the following conditions were used: acceleration voltage, 15 kV; and emission of current, 12 μA.

### 2.8 X-ray diffraction analysis of Cd in soil

X-ray diffraction (XRD) was performed for crystalline soil after the plant post-harvesting process to detect the total quantity of Cd left in the soil. During XRD, the sample was placed on an XRD grid and spectra were recorded at 40 keV and 30 mA, with Cu K-α radiation.

### 2.9 Statistical analysis

Statistical analysis of univariate data, including morphological and physiological characteristics of plants, was performed in RStudio software (version 4.2; The R Foundation for Statistical Computing, Vienna, Austria). Sharpio test (default function in R) was used for data normality. Normalized data were analyzed by constructing a two-way ANOVA, which was followed by the *post hoc* Tukey’s honestly significant difference (HSD) test (Tukey’s HSD, *p*< 0.005). Correlation analysis among different variables was performed with RStudio by identification of means, medians, and standard deviations (SDs). Following ANOVA and Tukey analysis, the data bars were labeled with mean and SD using the RStudio package “multcompLetters4”). Pearson’s correlation analysis was performed for a root architecture parameter and for analyzing morphophysiological parameters at maturity and at the early growth stage (i.e., early stage). XRD peak analysis was performed using X’Pert HighScore (version 3.0; Malvern Panalytical Ltd, Cambridge, UK) and graphs were made in RStudio software.

## 3 Results

### 3.1 Combinations enhanced growth and biomass under Cd stress

**Figure 1:**
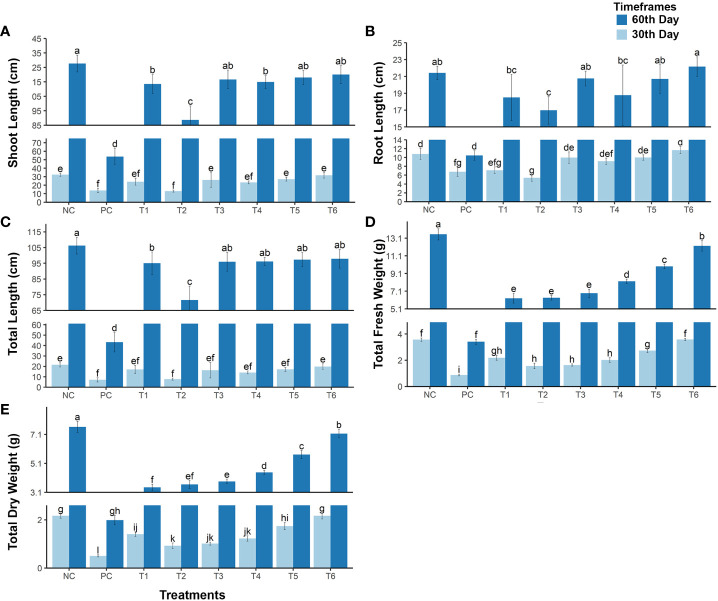
The growth rate of mustard plants grown under cadmium stress . The plants were analyzed at maturity and at an early stage of plant growth. **(A)** shoot length; **(B)** root length; **(C)** total length of the plant; **(D)** total fresh weight of the plant; and **(E)** total dry weight of the plant. The treatments are as follows: NC, negative control—mustard plant only; PC, positive control—mustard plant + cadmium; T1, treatment 1—Rhizobium leguminosarum (RL); T2, treatment 2—biochar (BC); T3, treatment 3—RL + BC; T4, treatment 4—Vigna radiata (VR); T5, treatment 5—VR + RL; T6, treatment 6—VR + BC + RL. Data present the interaction between plant growth and the applied cadmium stress. Significance was calculated using two-way ANOVA under the Tukey’s HSD post hoc test for normally distribution data (honestly significant difference, p < 0.005). HSD, Tukey’s honestly significant difference. The lower case letters show the significance difference between treatments.

The *post hoc* Tukey’s HSD test (*p*< 0.005) was used to analyze plant growth and biomass data. The results show that Cd stress significantly reduced shoot, root, and total plant length. Compared with normal conditions (i.e., the NC treatment), shoot, root, and total plant length in the PC treatment were, respectively, 59.33%, 52.79%, and 57.95% lower at maturity and 67.27%, 41.87%, and 57.21% lower in the early growth stage (see **Figures 1A–C**). However, different treatment combinations reduced or eliminated the effect of Cd stress and enhanced plant growth. Treatment T1 [i.e., *R. leguminosarum* only (RL)] reduced Cd stress, enhancing shoot, root, and total plant length by 48.78%, 36.24%, and 46.88%, respectively, at maturity, and by 46.38%, 2.85%, and 31.90%, respectively, in the early stage of growth. Treatment T2 [i.e., biochar only (BC)] was less effective than the other treatments, increasing shoot, root, and total plant length by only 26.81%, 29.54%, and 27.4%, respectively, at maturity, and by 3%, –12%, and –2%, respectively, in the early stage of plant growth (see **Figures 1A–C**).

The T3 treatment [i.e., the combination of *R. leguminosarum* (RL) and biochar (BC)] increased shoot, root, and total plant length under Cd stress by 49.62%, 46.38%, and 49.35%, respectively, at maturity, and by 42.89%, 27.36%, and 38.45%, respectively, in the early growth stage (see **Figures 1A–C**).

The T4 treatment [i.e., *V. radiata* only (VR)] enhanced shoot, root, and total plant length under Cd stress in the maturity stage by 49.8%, 37.51%, and 48.02%, respectively, and in the early stage of growth by 32.9%, 20.4%, and 29.3%, respectively. The T5 treatment (i.e., the combination of RL and VR) improved the growth of plants under Cd stress compared with the positive control (PC) in the maturity stage by 50.9%, 46.1%, respectively, and 50.4%, and in the early growth stage by 46.7%, 27.7%, and 41.14%, respectively (see **Figures 1A–C**). In contrast, the T6 treatment (i.e., the combination of VR, BC, and RL) enhanced plant growth to the same extent as the negative control (NC). This combination eradicates the effect of Cd stress, with shoot, root, and total plant length being, respectively, 92%, 98%, and 94.0% of that achieved under normal conditions in the maturity stage and 91%, 99%, and 97.7% of normal, respectively, in the early growth stage. This shows that the T6 treatment (i.e., the combination of VR, BC, and RL) effectively reduces Cd stress and improves plant growth (see **Figure 1**).

The presence of Cd significantly reduced plant fresh and dry biomass in the PC treatment, by 74.82% and 73.90%, respectively, in the mature stage and by 75.61% and 76.76%, respectively, in the early growth stage. However, exposure of mustard plants under Cd stress to different treatment combinations helps to increase the biomass of plants and decrease the toxic effect of Cd (see **Figures 1D, E**). The T1 treatment increased fresh and dry plant biomass by 21.35% and 19.10%, respectively, at maturity, and by 36.90% and 41.85%, respectively, in the early growth stage. The T2 treatment resulted in an increase in fresh and dry biomass of 21.74% and 21.72%, respectively, in the maturity stage, and of 19.30% and 19.49%, respectively, in the early growth stage. The results of T3 treatment were approximately 3% better than those of T2, with increases in fresh and dry biomass of 25.54% and 24.46%, respectively, being recorded in the maturity stage, and of 21.30% and 23.61%, respectively, in the early growth stage (see **Figures 1D, E**). The T4 treatment (i.e., VR co-planting) increased fresh and dry plant biomass by 35.53% and 32.63%, respectively, at the maturity growth stage, and by 31.93% and 33.55%, respectively, at the early growth stage. Even better results were observed with the T5 treatment (i.e., the combination of RL + VR) in both the mature (fresh and dry biomass increased by 47.97% and 48.90%, respectively) and early growth stages (increases of 52.11% and 57.25%, respectively). The greatest biomass enhancement was noted in the T6 treatment (i.e., the combination of RL + VR + BC). This treatment increased the fresh and dry biomass of mustard plants at maturity by 65.18% and 67.87%, respectively, and in the early stages of plant growth by 75.74% and 76.98%, respectively (see **Figures 1D, E**).

In addition, some other morphological traits were analyzed (see **Table 1**), namely dry and fresh weights of leaves, roots, and shoots in the early and maturity stages of growth. Differences in these traits between the negative control (NC) and the T6 treatment were also non-significant, although a significant difference between the positive control (PC) and other treatments was found. Therefore, the results confirm that a combination of bacteria, biochar, and co-planting improves plant biomass and significantly diminishes Cd toxicity.

**Table 1:** Characteristics of mustard plants grown under cadmium stress in the early and maturity stages of growth.

Treatment	Growth stage	LFW (g)	LDR (g)	RFW (g)	RDW (g)	SFW (g)	SDW (g)
**NC**	*Maturity*	3.97 ± 0.19^a^	1.93 ± 0.13^a^	4.37 ± 0.5^a^	2.62 ± 0.15^a^	4.44 ± 0.19^a^	2.64 ± 0.24^a^
	*Early*	1.34 ± 0.1^d^	0.81 ± 0.06^d^	0.85 ± 0.07^e^	0.57 ± 0.05^f^	1.58 ± 0.07^de^	0.79 ± 0.03^cd^
**PC**	*Maturity*	2.49 ± 0.36^c^	0.51 ± 0.09^ef^	1.28 ± 0.24^d^	0.87 ± 0.15^e^	1.1 ± 0.11^f^	0.57 ± 0.06^de^
	*Early*	0.67 ± 0.09^d^	0.14 ± 0.03^h^	0.26 ± 0.03^g^	0.17 ± 0.02^i^	0.42 ± 0.03^h^	0.2 ± 0.02^f^
**T1**	*Maturity*	3.35 ± 0.34^ab^	1.03 ± 0.11^c^	1.49 ± 0.06^d^	1.13 ± 0.08^d^	1.91 ± 0.11^bcd^	0.95 ± 0.05^c^
	*Early*	0.84 ± 0.05^d^	0.57 ± 0.06^e^	0.55 ± 0.05^efg^	0.43 ± 0.07^fgh^	0.83 ± 0.13^fg^	0.41 ± 0.06^ef^
**T2**	*Maturity*	3.52 ± 0.76^ab^	1.10 ± 0.12^c^	2.39 ± 0.11^c^	1.53 ± 0.17^c^	1.98 ± 0.21^bc^	0.99 ± 0.11^bc^
	*Early*	0.71 ± 0.09^d^	0.32 ± 0.04^fgh^	0.46 ± 0.03^g^	0.28 ± 0.05^hi^	0.65 ± 0.18^gh^	0.33 ± 0.09^ef^
**T3**	*Maturity*	2.92 ± 0.77^bc^	1.62 ± 0.10^b^	1.49 ± 0.06^d^	1.16 ± 0.03^d^	1.45 ± 0.36^e^	0.72 ± 0.18^cd^
	*Early*	0.66 ± 0.08^d^	0.31 ± 0.07^gh^	0.51 ± 0.04^fg^	0.36 ± 0.03^ghi^	0.68 ± 0.06^gh^	0.34 ± 0.03^ef^
**T4**	*Maturity*	2.84 ± 0.52^bc^	1.71 ± 0.13^b^	2.33 ± 0.14^c^	1.52 ± 0.11^c^	1.74 ± 0.14^cde^	0.87 ± 0.07^c^
	*Early*	0.81 ± 0.08^d^	0.46 ± 0.1^efg^	0.47 ± 0.02^g^	0.32 ± 0.02^hi^	0.89 ± 0.15^fg^	0.44 ± 0.08^ef^
**T5**	*Maturity*	3.79 ± 0.41^a^	1.95 ± 0.10^a^	3.28 ± 0.07^b^	2.18 ± 0.09^b^	2.22 ± 0.11^b^	1.23 ± 0.25^b^
	*Early*	1.11 ± 0.13^d^	0.77 ± 0.1^d^	0.54 ± 0.03^efg^	0.42 ± 0.07^fgh^	1.09 ± 0.05^f^	0.55 ± 0.02^de^
**T6**	*Maturity*	3.87 ± 0.29^a^	1.98 ± 0.06^a^	3.40 ± 0.23^b^	2.32 ± 0.15^b^	4.33 ± 0.27^a^	2.5 ± 0.28^a^
	*Early*	1.18 ± 0.17^d^	0.82 ± 0.05^d^	0.83 ± 0.05^ef^	0.55 ± 0.12^fg^	1.58 ± 0.07^de^	0.79 ± 0.04^cd^

LFW, leaf fresh weight; LDR, leaf dry weight; NC, negative control; PC, positive control; RFW, root fresh weight; RDW, root dry weight; SFW, shoot fresh weight; SDW, shoot dry weight.

Different lowercase letters in the same column indicate the significant difference between maturity and early stages of plant growth in the various treatments (p< 0.005).

### 3.2 The outcome of combinations on plant physiological parameters

**Figure 2:**
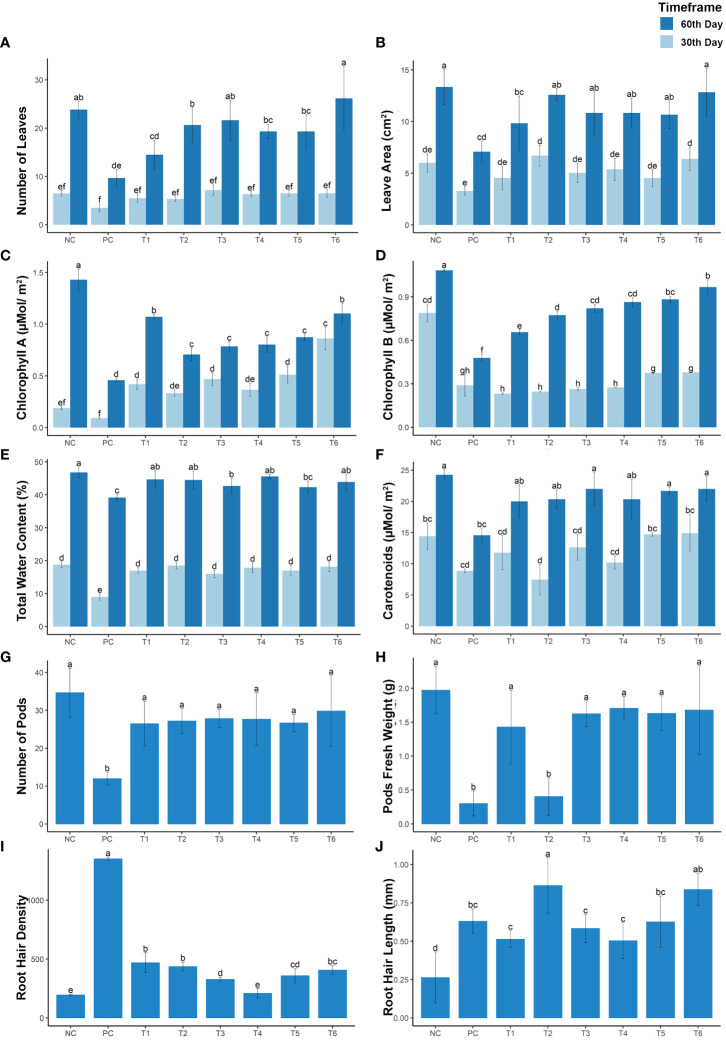
The growth rate of mustard plants grown under cadmium stress. The plants were analyzed at maturity and at an early stage of plant growth. The graphs present the following: **(A)** number of leaves; **(B)** leaf area; **(C)** chlorophyll a content; **(D)** chlorophyll b content; **(E)** total water content; **(F)** carotenoid content; **(G)** number of pods; **(H)** pod fresh weight; **(I)** root hair density; and **(J)** root hair length. The treatment are as follows: NC, negative control—mustard plant only; PC, positive control—mustard plant + cadmium; T1, treatment 1—Rhizobium leguminosarum (RL); T2, treatment 2—biochar (BC); T3, treatment 3—RL + BC; T4, treatment 4—Vigna radiata (VR); T5, treatment 5—VR + RL; T6, treatment 6—VR + BC + RL. Data present the interaction between plant growth and the applied cadmium stress. Significance was calculated using two-way ANOVA under the Tukey’s HSD post hoc test for normally distribution data (honestly significant difference, p < 0.005). HSD, Tukey’s honestly significant difference.

Physiological parameters of the mustard plant were analyzed, and differences were considered significant at *p*< 0.005. The parameters measured included the number of leaves, leaf area, chlorophyll a content, chlorophyll b content, total water content, and carotenoid content. These parameters were significantly reduced in treatment PC (under Cd stress), by 63.06%, 46.89%, 67.90%, 55.67%, 39.93%, and 16.36%, respectively, in the mature growth stage and by 51.19%, 50.95%, 89.47%, 63.30%, 40.57%, and 52.34%, respectively, in the early growth stage (see **Figures 2A–F**). However, different treatment combinations resulted in dramatic increases in these physiological parameters under Cd stress. For example, compared with the positive control (PC), the T1 treatment increased these physiological attributes by 18.47%, 20.66%, 42.81%, 16.46%, 22.40%, and 11.74%, respectively, at the mature growth stage and by 27.90%, 18.86%, 38.23%, –7.29%, 19.57%, and 42.86%, respectively, at the early growth stage. The T2 treatment, like other treatment combinations, was associated with a positive response to Cd stress, increasing the listed parameters by 42.03%, 41.29%, 17.32%, 27.32%, 23.78%, and 11.38%, respectively, at the mature growth stage and by 25.57%, 50.95%, 28.26%, –5.54%, –9.48%, and 50.85%, respectively, at the early stage of plant growth. However, the T3 treatment (i.e., the combination of RL with BC) increased the number of leaves, leaf area, chlorophyll a content, chlorophyll b content, total water content, and carotenoid content by 45.85%, 28.12%, 22.83%, 31.66%, 30.65%, and 7.47%, respectively, at maturity and by 51.14%, 26.07%, 43.95%, –3.26%, 25.31%, and 37.54%, respectively, in the early stage of growth. The T4 treatment (i.e., co-planting with VR) enhanced these physiological parameters under Cd stress by 36.94%, 28.16%, 24.01%, 35.66%, 23.78%, and 13.64%, respectively, at the mature growth stage and by 39.52%, 31.05%, 31.95%, –1.93%, 8.96%, and 47.30%, respectively, at the early growth stage. The same trend was observed in treatment T5 (i.e., the combination of RL + VR), which increased these parameters by 36.94%, 26.85%, 29.01%, 37.44%, 29.28%, and 6.72%, respectively, at the maturity stage of growth and by 41.85%, 18.36%, 49.05%, 10.75%, 39.19%, and 42.86%, respectively, at the early stage of growth. In plants subjected to treatment T6 (i.e., the combination of VR, BC, and RL), all parameters showed the same trends as of the negative control (NC) group, without Cd stress. These results show that these combinations significantly improve plant growth parameters in comparison with normal growth conditions. Under Cd stress, the T6 treatment (i.e., the combination of VR, BC, and RL) resulted in a remarkable increase in the number of leaves, leaf area, chlorophyll a content, chlorophyll b content, total water content, and carotenoid content, by 63.05%, 43.16%, 45.00%, 45.22%, 30.65%, and 10.14%, respectively, at the mature stage of growth and by 41.85%, 46.22%, 89.67%, 11.34%, 40.57%, and 49.07%, respectively, at the early stage of growth. Thus, the T8 combination treatment demonstrated the equivalent enhancement in plant attributes as compared with the negative control (NC). This result demonstrates that the combination of VR, BC, and RL significantly impacts plant growth and physiological parameters (see **Figures 2A–F**).

### 3.3 Fresh pod yield and root hair density

In the current study, the different treatment combinations provide a sustainable enhancement in yield quantity and weight. These parameters were increased by 41.82% and 57.19%, respectively, under treatment T1, by 43.75% and 5.16%, respectively, under treatment T2, and by 45.67% and 67.09%, respectively, under treatment T3. Treatment T4 increased quantity and growth by 45.19% and 71.24%, respectively. whereas treatment T5 (i.e., the combination of RL + VR) increased the yield and growth by 42.31% and 67.43%, respectively. However, treatment T6 increased the yield and growth of plants grown under Cd stress by 51.44% and 69.80%, respectively. These results corroborate the effectiveness of combinations of VR, BC, and RL in improving growth and yield enhancement when plants are grown under Cd stress (see **Figures 2G, H**).

Plant roots are the first organs that interact with Cd in the soil. Cd stress induces significant changes in mustard plant roots by increasing the length and density of the root hairs. In this study, large increases in root hair length and density were observed in PC plants at the early growth stage. The rate of enhancement varied between treatments. Root hair growth and density were reduced by 28.95% and 20.26%, respectively, by the T1 treatment, by 69.49% and 17.90%, respectively, by the T2 treatment, by 37.06% and 9.86%, respectively, by the T3 treatment, by 27.79% and 1.01%, respectively, by the T4 treatment, and by 42.08% and 12.14%, respectively, by the T5 treatment. The T6 treatment (i.e., the combination of VR, BC, and RL) reduced the root hair length and density by 66.40% and 15.71%, respectively. This result concludes that these combinations provide potential to plant for its suitable growth under Cd stress (see **Figures 2I, J**).

### 3.4 Cd detection in leaves and roots with SEM/EDX

**Figure 3:**
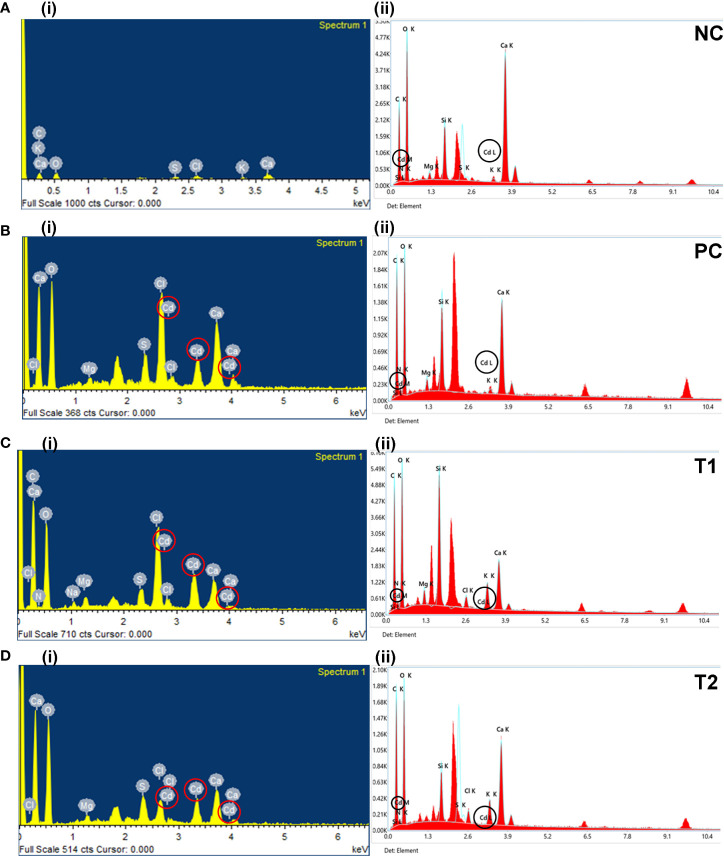

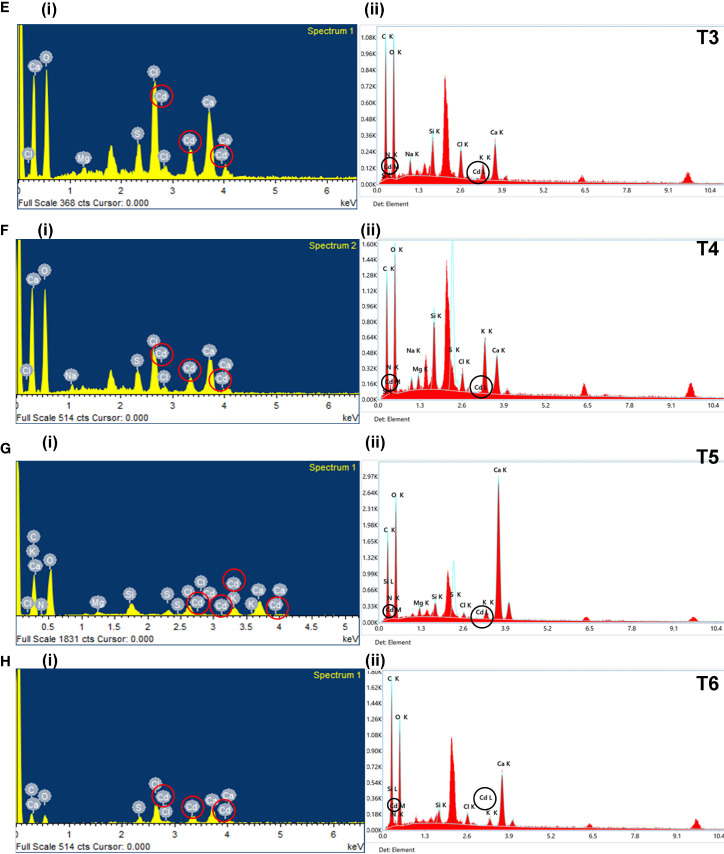
The EDX graphs for leaf (i) and root (ii) tissue for all treatments (i.e., NC, PC, T1, T2, T3, T4, T5, and T6). The treatments are as follows: NC, negative control—mustard plant only; PC, positive control—mustard plant + cadmium; T1, treatment 1—*Rhizobium leguminosarum* (RL); T2, treatment 2—biochar (BC); T3, treatment 3—RL + BC; T4, treatment 4—*Vigna radiata* (VR); T5, treatment 5—VR + RL; T6, treatment 6—VR + BC + RL. EDX, energy-dispersive X-ray.

The analysis of dried leaves and root samples by EDX confirmed the presence of Cd ions in the leaves and roots of plants in the maturity stage (**Figure 3**). **Table 2** shows the percentage by weight of different macroelements, including Cd, in the mustard plants. The results not only confirm the presence of Cd in root and leaf tissue but also show that different treatment combinations help to reduce Cd uptake, thereby increasing plant growth and nutrient uptake. The EDX results show that Cd in roots and leaves reduces nutrient absorption (see **Table 2**). The presence of high concentrations of Cd can interfere with a plant’s uptake of essential minerals, leading to reduced growth and yield.

**Table 2:** The weight percent (W%) and atomic percent (A%) of macroelements present in dried leaf and root samples of different treatments.

Element	NC				PC				T1				T2			
	*Leaves*		*Root*		*Leaves*		*Root*		*Leaves*		*Root*		*Leaves*		*Root*	
	*W%*	*A%*	*W%*	*A%*	*W%*	*A%*	*W%*	*A%*	*W%*	*A%*	*W%*	*A%*	*W%*	*A%*	*W%*	*A%*
**C**	30.22	39.9	16.5	25	40.1	53	23.5	32.7	45	55.8	31	41.1	–	–	31	42.3
**N**	8.5	9.62	4.2	5.3	2.3	2.6	5.1	6.1	1.7	1.81	5	5.7	–	–	3.6	4.3
**O**	43.42	43	50.2	56	38.4	38	48.2	50.2	38.9	36.3	43.5	43.3	72.66	87	41.1	42.2
**Na**	–	–	–	–	–	–	–	–	0.45	0.29	–	–	–	–	–	–
**Mg**	–	–	0.8	0.6	–	–	1.4	1	0.81	0.5	1.4	0.9	1.55	1.22	–	–
**Si**	–	–	4	2.5	–	–	6.7	4	–	–	8.2	4.6	–	–	3	1.8
**S**	2.29	1.13	4.5	2.5	–	–	–	–	1.44	0.67	–	–	5.07	3.03	7.5	3.8
**Cl**	4.24	1.9	–	–	8.05	3.6	–	–	7.99	3.36	1.1	0.5	5.62	3.04	0.2	–
**K**	1.81	0.73	0.4	0.2	–	–	0.5	0.2	–	–	2.9	1.2	–	–	1	0.5
**Ca**	9.52	3.68	19.3	8.5	4.72	1.9	14.1	5.9	3.25	1.21	6.8	2.7	10.33	4.93	2.4	1
**Cd**	0.3	0	0.1	0	6.44	0.9	0.5	0.1	0.45	0.06	0.1	0	4.76	0.81	10.2	4.2
	**T3**				**T4**				**T5**				**T6**			
	***Leaves***		***Root***		***Leaves***		***Root***		***Leaves***		***Root***		***Leaves***		***Root***	
	*W%*	*A%*	*W%*	*A%*	*W%*	*A%*	*W%*	*A%*	*W%*	*A%*	*W%*	*A%*	*W%*	*A%*	*W%*	*A%*
**C**	–	–	34	44	71.7	86	33.3	45	42.8	53.1	18.9	28.3	41.17	56.3	34.9	44.7
**N**	–	–	5.2	5.7	–	–	4.9	5.7	2.89	3.08	3.1	3.9	–	–	4.4	4.8
**O**	63.2	80.4	44.6	43	–	–	36.4	36.9	46.4	42.1	46.9	52.7	31.87	32.7	46.4	44.7
**Na**	–	–	2.7	1.8	1.56	1.3	1.3	0.9	–	–	–	–	–	–	–	–
**Mg**	1.02	0.85	–	–	–	–	1	0.6	0.55	0.34	1.1	0.8	–	–	–	–
**Si**	–	–	2.9	1.6	–	–	3.6	2.1	1.51	0.8	1.3	0.8	–	–	1.7	0.9
**S**	3.96	2.51	–	–	4.13	2.5	9.1	4.6	0.95	0.44	4.8	2.7	2.79	1.43	–	–
**Cl**	15.47	8.88	2.8	1.2	9.82	5.3	1.3	0.6	2.21	0.93	0.5	0.3	10.97	5.08	1.4	0.6
**K**	–	–	1.8	0.7	–	–	4.6	1.9	2.02	0.77	1	0.5	–	–	1.1	0.4
**Ca**	13.63	6.92	5.4	2.1	10.5	5	4.1	1.7	0.13	0.02	22.2	9.9	9.83	4.03	9.9	3.8
**Cd**	2.72	0.49	0.4	0.1	2.4	0.4	0.2	0	4.23	1.57	0.2	0	3.37	0.49	0.2	0

The overall W% and A% are equivalent to 100 in each column.

NC, negative control; PC, positive control.

In addition, Cd toxicity may also cause damage to other organs, leaves. **Table 2** shows that treatment T6 was associated with the lowest Cd accumulation in leaves and roots (3.37% and 0.2%, respectively). However, no changes were observed in the morphophysiological features of the plants. In the comparison of roots and leaves, the EDX analysis of all treatments demonstrated that Cd accumulation in leaves was higher than in roots. This result confirms that different combinations reduce Cd around the roots in soil. In addition, plants subjected to treatment T2 (i.e., BC only) showed considerable Cd at the root level. This might be because the absorption of Cd by BC was lower when BC was used alone than when it was part of combination treatments. A comparison of morphophysiological parameters in **Figures 1**, **2** shows that the results of treatment T2 were not as good as those obtained with the other treatments.

**Figure 4:**
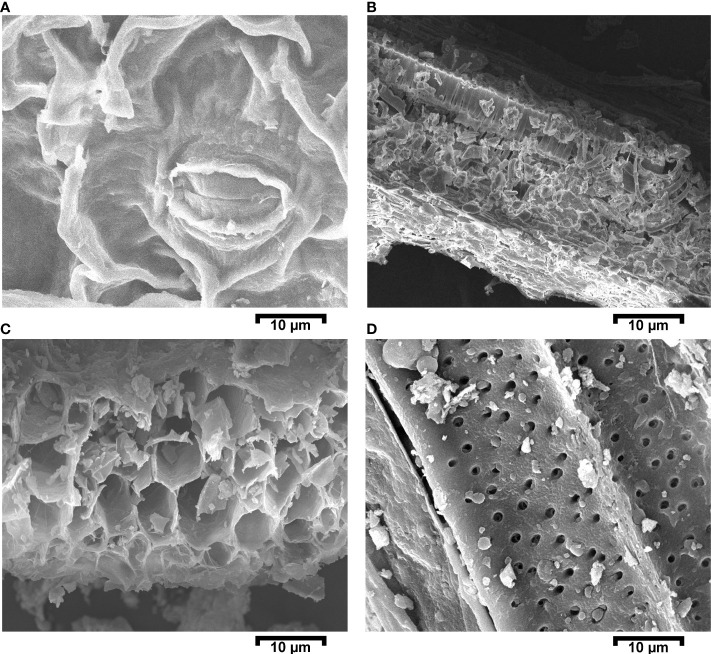
Scanning electron micrograph images for the positive control (PC) (i.e., a mustard plant grown under cadmium stress. Images were captured on a 10-µm scale. **(A)** Leaf with a closed stomata; **(B)** cross-sectional view of a leaf; **(C)** cross-sectional view of a primary root; and **(D)** upper structural primary root.

The effect of Cd on leaf stomata, mesophyll cells, and primary roots was studied *via* SEM on the 60th day of growth. **Figure 4** shows that Cd in plants exposed to the PC treatment caused a significant number of stomata to be closed. Stomatal closure prevents water transpiration, which in turn leads to a reduction in nutrient loss from the plant, thus making more nutrients available in the long term. Some irregular structures were observed in mesophyll cells and attributed to Cd accumulation. In addition, the presence of Cd affected the internal and external structures of the primary roots. Cd accumulation reduces the translocation of other minerals *via* root hairs and inhibits the growth of the germ tissue region. We observed thinning and breakage of the root cell compartments, and an excessive number of pores on root hairs (see **Figures 4A–D**).

### 3.5 Identification of Cd in soil samples after harvesting

**Figure 5:**
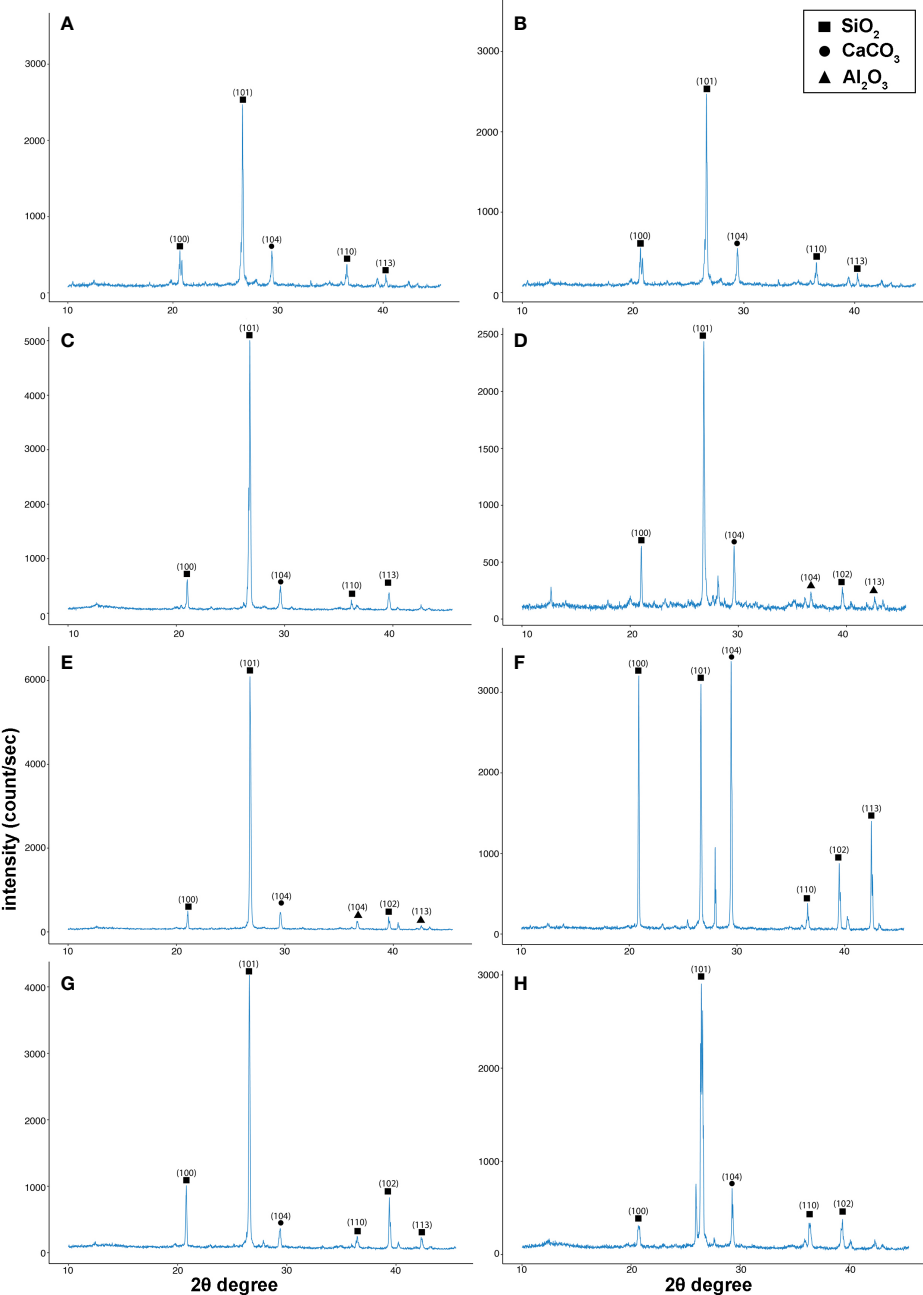
X-ray diffraction (XRD) graphs for soil analysis identifying the different compounds present in the soil. **(A)** negative control group (NC), **(B)** positive control group (PC), **(C)** as T1, **(D)** as T2, **(E)** as T3, **(F)** as T4, **(G)** as T5, and **(H)** as T6. The treatments are as follows: NC, negative control—mustard plant only; PC, positive control—mustard plant + cadmium; T1, treatment 1—*Rhizobium leguminosarum* (RL); T2, treatment 2—biochar (BC); T3, treatment 3—RL + BC; T4, treatment 4—*Vigna radiata* (VR); T5, treatment 5—VR + RL; T6, treatment 6—VR + BC + RL.

Soil samples were collected after harvesting plants at maturity for the analysis of abandoned Cd traces in the soil. XRD was performed and the analysis was within a scanning range of 10°–50° (2θ). Five distinct diffraction peaks were found, at 20.84°, 26.62°, 29.42°, 36.46°, 39.44°, and 42.47°, in all eight treatments, with indexed as the planes 100, 101, 104, 110, 102, and 113, respectively, as shown in **Figure 5**. Three different chemicals were observed in the XRD analysis: SiO_2_, CaCO_3_, and Al_2_O_3_. However, no evidence of CdCl_2_ was found in the post-harvested soil. This confirms that *B. juncea* finished the phytoremediation process and cleaned the soil. In contrast to the EDX results, Cd was detected in roots and leaves. However, with different co-combination treatments Cd concentration in the root and leaves of the mustard plant indicates that co-combination treatments inhibit the translocation of the Cd as compared with positive control (PC). The results confirmed that the T6 treatment (i.e., the combination of BC, VR, and RL) helps to reduce Cd uptake in mustard plants and performs a phytoremediation function. In addition, as the mustard plant performs phytoremediation, the combination reduces the translocation of Cd in the plant. These treatments will be effective for non-hyperaccumulating plants.

### 3.6 Correlation of root traits and architecture

**Figure 6:**
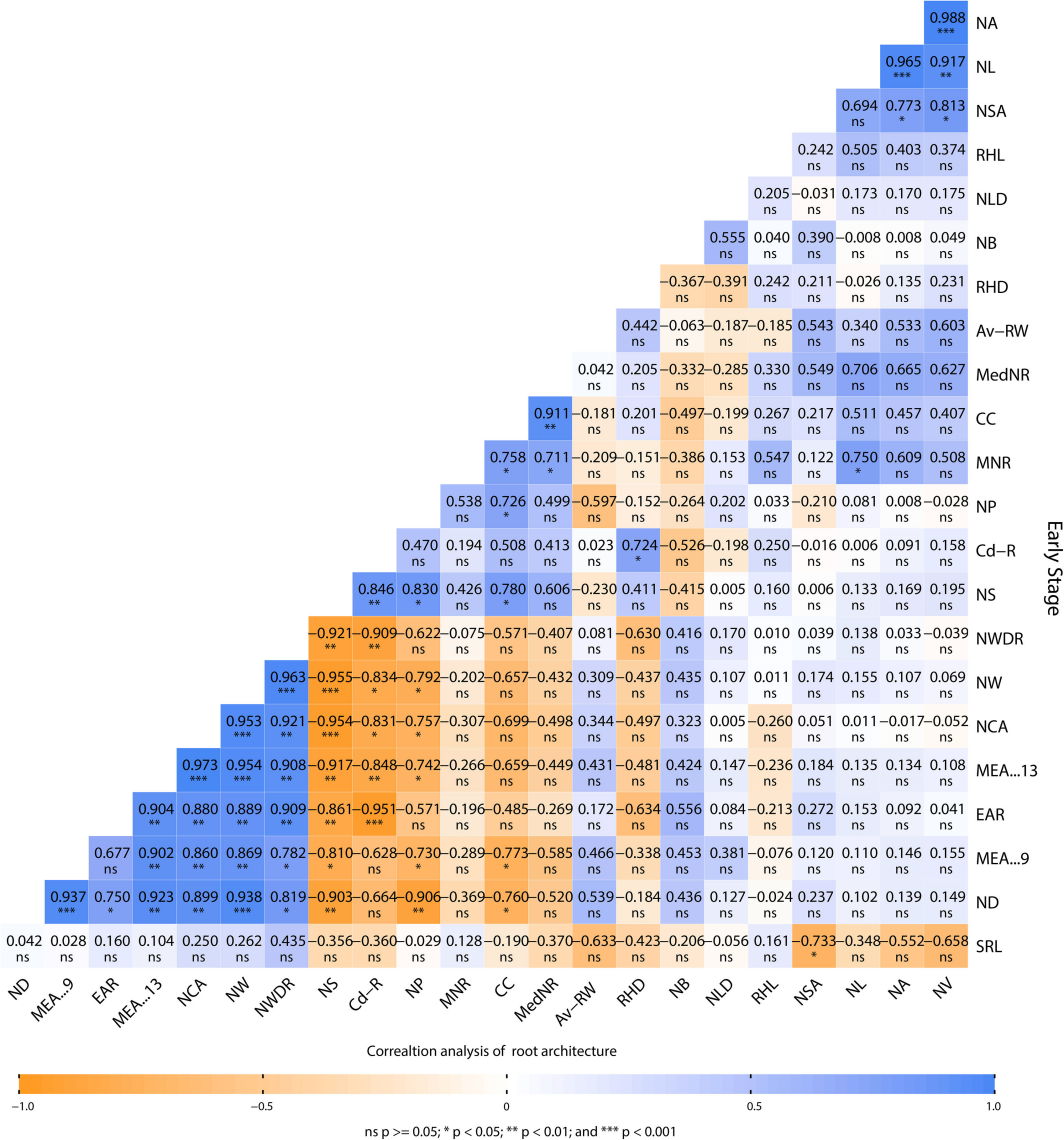
Pearson’s correlation performed on root architecture attributes of Brassica juncea grown in cadmium-contaminated soil. Av-RW, average root width (diameter); NB, network bushiness; CC, connected components; ND, network depth; EAR, ellipse–axes ratio; NLD, network length distribution; MEA-13, major ellipse axis; MNR, maximum number of roots; NW, network width; MedNR, median number of roots; MEA-9, minor ellipse axis; NA, network area; NCA, network convex area; NP, network perimeter; NS, network solidity; SRL specific root length; NSA, network surface area; NL, network length; NV, network volume; NWDR, network width-to-depth ratio; Cd-R, cadmium in the root; RHD, root hair density; RHL, root hair length.

Root architecture traits were evaluated under Cd stress in the early growth stage of the plant (i.e., at 30 days). The root architecture data were analyzed through the GiA Roots software to determine the different root traits. Data were further analyzed through Pearson’s correlation analysis in RStudio to examine the positive and negative correlation among the different root traits. The correlation of (Cd-R), NS, NP, MNR, CC, MedNR, and RHD show a negative correlation with SRL, ND, MEA-9, EAR, MEA-13, NCA, NW, NWDR, NSA, NLD, and NB (**Figure 6**). We confirmed that the following traits:- NS, NP, MNR, CC, MedNR, RHD, SRL, ND, MEA-9, EAR, MEA-13,NCA, NW, NWDR, NSA, NLD, and NB decrease when plants are grown in the presence of Cd stress. Cd-R shows a positive correlation with NV, NA, NL, RHL, RHD, Av-RW, MedNR, CC, MNR, and NP, confirming that Cd enhanced these traits in the root system. However, NP, MNR, CC, MedNR, and RHD showed a positive correlation with NV, NA, NL, RHL, RHD, and other parameters. This demonstrates that Cd presence enhances some root traits and reduces others.

### 3.7 Correlation of early and maturity stages of plant

**Figure 7:**
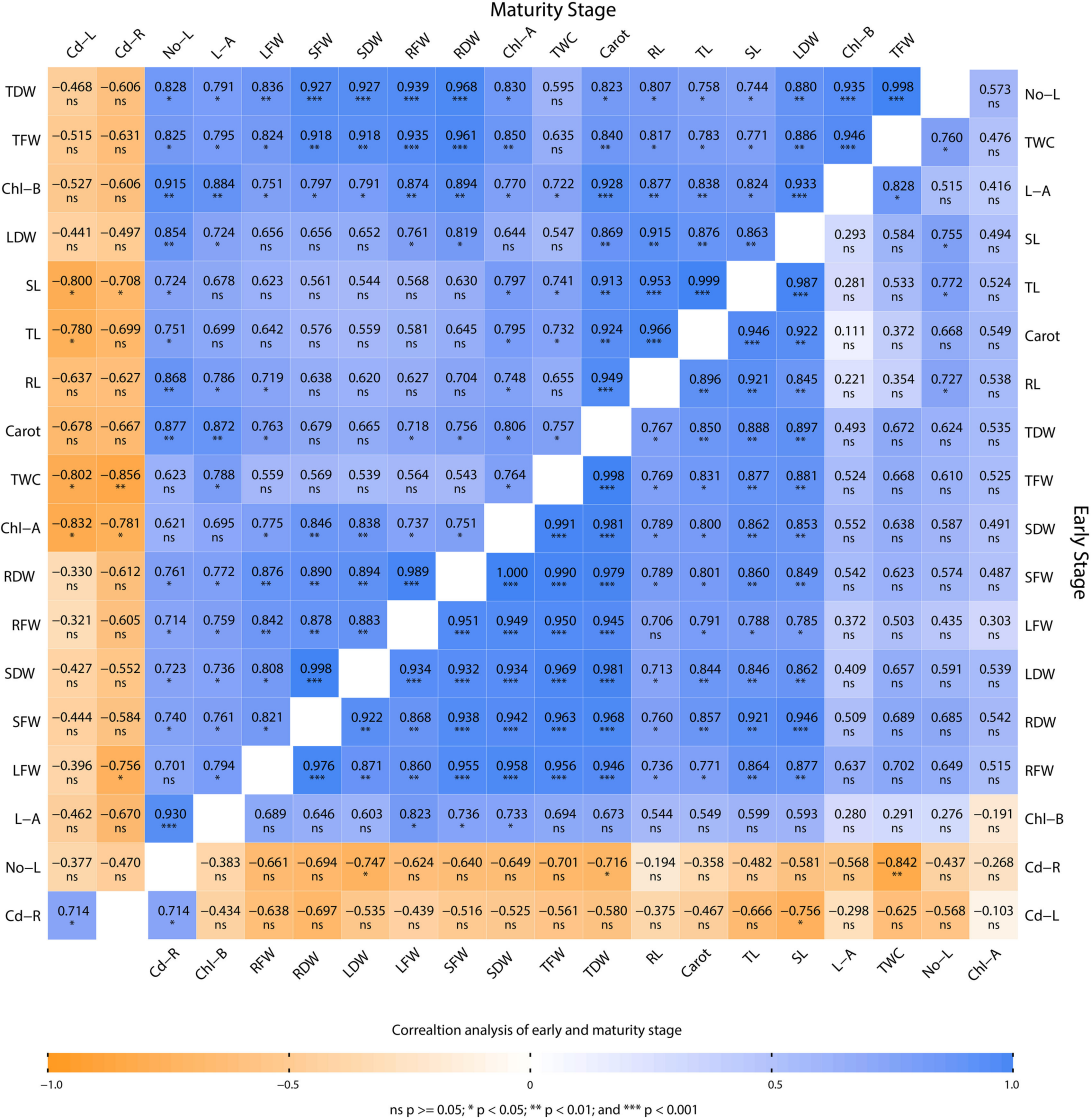
Pearson’s correlation performed on the various morphophysiological attributes of Brassica juncea grown in Cd-contaminated soil. The plants were analyzed at maturity and at an early stage of plant growth. TL, total length; SL, shoot length; RL, root length; SFW, shoot fresh weight; RFW, root fresh weight, LFW, leaf fresh weight; TFW, total fresh weight; Cd-L cadmium in leaves; SDW, shoot dry weight; RDW, root dry weight; LDW, leaf dry weight; TDW, total dry weight; Cd-R, cadmium in root; No-L, number of leaves; L-A, leaf area; Chl-A, chlorophyll a content; Chl-B, chlorophyll b; carot, carotenoid content; TWC, total water content.

Pearson’s correlation analysis was used to investigate the relationship between various morphophysiological parameters of mustard plants grown under Cd stress and different combinations of biochar, *V. radiata*, and *R. leguminosarum* (**Figure 7**). The correlation analysis was carried out at two time points, that is, at maturity (65 days) and at an early stage of growth (30 days). At both time points, Cd concentrations in the root (Cd-R) and leaves (Cd-L) show a negative correlation with TL, SL, RL, SFW, RFW, LFW, TFW, Cd-L, SDW, RDW, LDW, TDW, No-L, L-A, Chl-A, Chl-B, Carot, and TWC. Only Cd-R and Cd-L were positively correlated with each other. In addition, all physiological characteristics were positive correlated with each other. Hence, these results reveal a close connection between the presence of Cd and a reduction in morphophysiological characteristics.

## 4 Discussion

The increased prevalence of Cd contamination in soils is an emerging worldwide concern (Gill, 2014). Cd is a heavy metal that causes adverse effects in crops. In developing countries, the presence of Cd causes serious disturbances in the food chain. Food contaminants also negatively impact human and animal health (Haider et al., 2021). The main purpose of this study was to evaluate the combination of biochar, intercropping (*V. radiata)*, and *R. leguminosarum* on crops grown in Cd-contaminated soil. For this purpose, the mustard plant was selected because of its recognizable hyperaccumulation properties (Rizwan et al., 2016a). The mustard plant is highly consumed as a source of food and is of economic importance throughout the world. This plant is the third-largest oil production plant after palm oil and soybean (FAO, 2017). In the current study, Cd stress decreased plant growth, biomass, photosynthesis pigmentation, and yield; however, several studies reported in the literature have shown similar results. The observed reduction in growth, biomass, and photosynthesis indicates that Cd decreases plant nutrient uptake and alters the plant’s cellular structure (see **Figures 1**, **2**) (Rizwan et al., 2012; Ehsan et al., 2014; Rizwan et al., 2016b).

The use of biochar improves the stability of plant growth, photosynthesis, and yield, and induces defense mechanisms in plants grown under Cd stress (Pandit et al., 2018). The T2 (BC), T3 (BC + RL), and T6 (BC + RL + VR) treatments resulted in increased biomass, photosynthesis, and yield. Biochar contains a sustainable source of nutrients that helps to mitigate the effects of Cd and provides beneficial growth conditions to plants (Jang et al., 2018). The T2 treatment might also increase soil physiochemical properties to generate a better environment for plant growth (Sun et al., 2013). A study by Younis et al. (2016) found that 3% biochar enhances plant biomass and photosynthesis in *Spinacia oleracea* when it is grown under Cd stress. In our study, 1.2% biochar (i.e., T2 treatment) increased biomass growth by 19% at the early stage and by 21% at the mature stage. The photosynthesis pigmentation improved under the influence of biochar by 60% compared with the positive control (PC) (see **Figure 1**). In addition, the T2 treatment reduced fresh and dry weight loss compared with the PC. However, even more positive results were achieved using combinations of biochar and the intercropping plant *V. radiata* and *R. leguminosarum* (RL).

Inoculating plants with plant growth-promoting rhizobia (i.e., *R. leguminosarum*) stimulates growth and improves nutrient uptake in Cd-contaminated soils (Hao et al., 2014). *R. leguminosarum* forms a symbiotic relationship with the mustard plant. These bacteria developed a nitrogen-fixing symbiotic relationship with plants in sandy soil and enhance nutrient uptake. The bacteria repressed Cd translocation and promoted different defense mechanisms in the mustard plants that helped the plants to overcome Cd stress. A previous study, by Safronova et al. (2006), reported that pea plant biomass increased under Cd stress with the presence of *R. leguminosarum*. In our study, plant growth, biomass, photosynthetic pigments, yield quantity, and root parameters improved with *R. leguminosarum* inoculation in treatments T1, T3, T5, and T6 (see **Figure 2**). However, Cd and *R. leguminosarum* interact directly with the plant roots in the rhizosphere. The root is the tissue that directly interacts with bacteria, biochar, intercropping plants, and Cd. The roots of mustard plants grown in Cd-contaminated soil in the presence of *R. leguminosarum* did not show any significant change. Thus, *R. leguminosarum* might provide favorable conditions for root growth and development. A study conducted by Taye et al. (2022) found that the length and diameter of roots of *Brassica napus* plants grown in Cd-contaminated soil in the presence of *R. leguminosarum* increased until the flowering stage, then started to decrease until the plants reached maturity. In our study, significant changes in root length and diameter were not observed at the early stage of growth (i.e., at the 30th day), but were noted at the maturity stage (i.e., at the 60th day) (see **Figures 2I, J**). This confirms that the Brassicaceae family shows no changes in growth in response to Cd stress until the flowering stage, after which root growth becomes stunted (see **Figure 1B**). Nguyen (2009) and Taye et al. (2022) reported that the abundance of bacteria present has a positive correlation with root length. This might be attributable to causal symbiotic relationships between plants and bacteria. Rhizosphere microbes could bring about change in plant root architecture by providing phytohormones (Asghar et al., 2004). Our results show that *R. leguminosarum* (included in treatments T1, T5, and T6) promotes root growth in *B. juncea* in both the early and mature stages of growth (see **Figures 1**, **2**).

Cadmium inhibits root growth and development in many plants (Ďurčeková et al., 2007). *B. juncea* shows tolerance to Cd, as a result of its hyperaccumulation properties, and Dhaliwal et al. (2022) found that root dry biomass in plants grown under Cd stress was higher in *B. juncea* than in other *Brassica* species (e.g., *B. rapa* and *B. napus*). This suggests that *B. juncea* root is more Cd tolerant than other species. A previous study, conducted by Meyers et al. (2008), reported that *B. juncea* has a highly significant potential to sequester different HMs in the root tissue. In contrast, we found that root biomass was 30% lower in the positive control (PC) than in the negative control (NC). Although large increases in root biomass were observed in other treatments, confirming the positive impact of the combination treatments on the mustard plant. A similar trend toward a reduction in yield and biomass has been observed in different *Brassica* species (Dhaliwal et al., 2020). This plant tends to escape from stress by enhancing its root rhizosphere, and, therefore, root diameter, root hair density, and root hair length increase in plants grown under Cd stress (Dhaliwal et al., 2022). However, the analysis of different attributes of root architecture concluded that some attributes were enhanced by Cd contamination, whereas others were reduced (see **Figure 6**) (Staňová et al., 2012; Kuijken et al., 2015; Cordero et al., 2020).

A study carried out by Yuan et al. (2017) found that the intercropping of *B. rapa* with *Clinopodium confine* increased root, stem, leaf, and shoot biomasses, in contrast to our study, intercropping between *B. juncea* with *V. radiata* resulted in significant increases in biomass. Additionally, Wu et al. (2019) reported that *V. radiata* shows significant growth enhancement when intercropped with *C. betacea* under Cd stress. Therefore, it might be possible that *V. radiata* enhances intercropped plant growth by providing metabolic or symbiotic help to the co-plant (**Figures 1**, **2**).

The SEM/EDX results confirmed that Cd translocation takes place in leaf and root tissues (see **Figure 3**). This is due to the hyperaccumulation properties of the mustard plant, which stores heavy metal ions in its aerial parts. However, soil analysis *via* XRD confirms that the post-harvesting soil was free from Cd contamination (see **Figure 5**). The Cd concentration under different treatments varies in comparison with the positive control (PC), showing that different combinations help to prevent the translocation of Cd in the mustard plant to differing extents (see **Table 2**). The correlation analysis also proves the negative impacts of Cd on different morphophysiological attributes of mustard plants and root traits (see **Figures 6**, **7**). A study conducted by Saleem et al. (2022) reported similar results.

Our results show that the toxic effects of Cd on all morphophysiological attributes were reduced by combinations of treatments, especially a combination of *R. leguminosarum*, *V. radiata*, and biochar (treatment T6). This combination gives us a high percentage of yield, biomass, growth, pigmentations, and root traits at both time points (see **Figures 1**, **2**). Moreover, our results showed that treatment T6 (i.e., a combination of RL, VR, and BC) decreased the uptake of Cd in the roots and leaves of the mustard plant (see **Figures 5** **–** **7**). This might be due to the restriction of Cd translocation (by the combination of RL, VR, and BC) and a decrease in the concentration of free Cd ions in the rhizosphere (Seth et al., 2008; Younis et al., 2016; Tang et al., 2020).

## 5 Conclusion

From the current study, it can be concluded that mustard plants readily absorb Cd through their roots and that CD is subsequently translocated to the aerial parts of the plant, where it accumulates. Cd prevents and reduces the quality of other mineral nutrients in the root tissue, which in turn causes disturbance in different processes within the plant. Accumulation of Cd in the leaves disturbs plant photosynthesis activity by reducing the quantity of chlorophyll a, chlorophyll b, and carotenoids, thus causing a reduction in the value of soil plant analysis development (SPAD) and photosynthesis activity. In addition, in the positive control treatment, SEM images show Cd toxicity, closed stomata, disturbed spongy mesophyll cells in the leaves, thinning and breakage in the root cell compartments, and an excessive number of pores on the root hairs. The closing of stomata in the presence of Cd restricted water translocation and CO_2_ uptake from the environment, and t disrupted the respiration and photosynthesis mechanisms of the plants. These findings prove that combinations of *V. radiata, R. leguminosarum*, and biochar, as in treatment T6 (BC + RL + VR), reduce the negative impact of Cd toxicity in plants, leading to increased growth, weight, photosynthesis pigment contents, yield quality, yield quantity, and water content, while reducing the translocation of Cd. Moreover, EDX confirmed that the T6 treatment (BC + RL + VR) reduced Cd translocation compared with the positive control (PC). The T6 treatment (BC + RL + VR) significantly prevented Cd accumulation in the plant’s root zone, reducing the further uptake of Cd ions and enhancing nutrient uptake. Furthermore, the present study identified that the T6 treatment combination alleviated Cd toxicity at an advanced stage of growth and improved root structure, nutrient mobility, photosynthesis, and growth. The T6 treatment (BC + RL + VR) reduced Cd toxicity at both time points, particularly at the 60 days maturity stage. We can conclude that treatment T6 (BC + RL + VR) influences plant growth temporarily during the early stages of plant growth (i.e., at 30 days), but that the response is permanent at the maturity stage. Plant morphophysiological attributes were enhanced by the T6 treatment, by up to 70%–95%. The T6 treatment resulted in a growth rate equivalent to that of the negative control (NC). Thus, the T6 treatment has been found to be the most effective combination to mitigate the effects of Cd contamination and should be used in the future. However, in future work, different intercropping plants, biochar products, and beneficial microbe combinations could be tested. In addition, long-term field studies should be conducted, and a parallel analysis of plants, root structures, and nutrient mobility under different HM stresses carried out, to gain further insights into the mechanisms underlying of the effects of these treatment combinations.

## Data availability statement

The original contributions presented in the study are included in the article/supplementary materials, further inquiries can be directed to the corresponding author.

## Author contributions

RAm and TS designed the project and scheme of work. Q-u-AH, MM, RAz, and RAm conducted the research. Q-u-AH collected and analyzed the data. Q-u-AH wrote the manuscript draft for submission. RAm, FM, AG, and AH reviewed the manuscript. All authors contributed to the article and approved the submitted version.
