# Corrigendum: An integrated remediation approach using combinations of biochar, *Rhizobium leguminosarum*, and *Vigna radiata* for immobilizing and dissipating cadmium contaminants from the soil–mustard plant system

**DOI:** 10.3389/fpls.2025.1627427

**Published:** 2025-05-30

**Authors:** Qurat-ul-Ain Ali Hira, Midhat Mahboob, Rimsha Azhar, Faiza Munir, Alvina Gul, Asim Hayat, Tariq Shah, Rabia Amir

**Affiliations:** ^1^ Department of Plant Biotechnology, Atta-ur-Rahman School of Applied Biosciences (ASAB), National University of Sciences and Technology (NUST), Islamabad, Pakistan; ^2^ Land Resource Research Institute, National Agricultural Research Center (NARC), Islamabad, Pakistan; ^3^ Department of Agronomy, Faculty of Crop Production Sciences, The University of Agriculture, Peshawar, Pakistan

**Keywords:** cadmium remediation, mustard plant, phytoremediation, biochar, intercropping, plant growth-promoting rhizobacteria (PGPR)

In the published article, there was an error in affiliation 3. Instead of “^3^Plant Science Research Unit, U.S. Department of Agriculture-Agricultural Research Service (USDA-ARS), Washington, DC, United States”, it should be “^3^Department of Agronomy, Faculty of Crop Production Sciences, The University of Agriculture, Peshawar, Pakistan”.

The authors apologize for this error and state that this does not change the scientific conclusions of the article in any way. The original article has been updated.

The U.S. Department of Agriculture has notified us that Tariq Shah was not and has never been an employee of the U.S. Department of Agriculture.

